# Potential effectiveness of local radiotherapy for extending survival and reducing symptomatic local events in patients with de novo metastatic prostate cancer

**DOI:** 10.1002/bco2.35

**Published:** 2020-08-30

**Authors:** Naoki Terada, Takashi Mizowaki, Toshihiro Saito, Akira Yokomizo, Naoki Kohei, Ken‐ichi Tabata, Masaki Shiota, Atsushi Takahashi, Toru Shimazui, Takayuki Goto, Yasuhiro Hashimoto, Masato Fujii, Ryotaro Tomida, Toshihiko Sakurai, Kohei Hashimoto, Sadafumi Kawamura, Shogo Teraoka, Shinichi Sakamoto, Takahiro Kimura, Manabu Kamiyama, Shintaro Narita, Nobumichi Tanaka, Takuma Kato, Masashi Kato, Takahiro Osawa, Takahiro Kojima, Takahiro Inoue, Mikio Sugimoto, Hiroyuki Nishiyama, Toshiyuki Kamoto, Japanese Urological Oncology Group

**Affiliations:** ^1^ Department of Urology Miyazaki University Miyazaki Japan; ^2^ Department of Radiation Oncology & Image‐applied Therapy Kyoto University Kyoto Japan; ^3^ Department of Urology Niigata Cancer Center Hospital Niigata Japan; ^4^ Department of Urology Harasanshin Hospital Fukuoka Japan; ^5^ Department of Urology Shizuoka General Hospital Shizuoka Japan; ^6^ Department of Urology Kitasato University Sagamihara Japan; ^7^ Department of Urology Kyushu University Fukuoka Japan; ^8^ Department of Urology Hakodate Goryoukaku Hospital Hakodate Japan; ^9^ Department of Urology Ibaraki Prefectural Central Hospital Ibaraki Cancer Center Kasama Japan; ^10^ Department of Urology Kyoto University Kyoto Japan; ^11^ Department of Urology Hirosaki University Hirosaki Japan; ^12^ Department of Urology Shikoku Cancer Center Matsuyama Japan; ^13^ Department of Urology Yamagata University Yamagata Japan; ^14^ Department of Urology Sapporo Medical University Sapporo Japan; ^15^ Department of Urology Miyagi Cancer Center Natori Japan; ^16^ Department of Urology Tottori University Yonago Japan; ^17^ Department of Urology Chiba University Chiba Japan; ^18^ Department of Urology Jikei University Tokyo Japan; ^19^ Department of Urology University of Yamanashi Hospital Chuo Japan; ^20^ Department of Urology Akita University Akita Japan; ^21^ Department of Urology Nara Medical University Kashihara Japan; ^22^ Department of Urology Kagawa University Kagawa Japan; ^23^ Department of Urology Nagoya University Nagoya Japan; ^24^ Department of Renal and Genitourinary surgery Hokkaido University Sapporo Japan; ^25^ Department of Urology University of Tsukuba Hospital Tsukuba Japan

**Keywords:** metastasis, prostate cancer, radiation therapy

## Abstract

**Objectives:**

To evaluate the association between the use of local radiotherapy (RT) with the survival of patients with de novo metastatic prostate cancer and symptomatic local events (SLEs).

**Patients and methods:**

Patients were initially diagnosed with metastatic prostate cancer between 2008 and 2017 at 30 institutes in Japan. Prostate‐specific antigen (PSA) progression‐free survival (PSA‐PFS) under initial androgen deprivation therapy and overall survival (OS) was compared between patients receiving local RT (RT group) and no RT (no‐RT group) by multivariate Cox proportional hazard analyses. The occurrence rate of grade ≥2 SLEs was compared by multivariate logistic regression analyses. Propensity score matching (PSM) analyses were performed to compare PSA‐PFS and OS of the groups in the high and low metastatic burden cohort.

**Results:**

Two hundred and five (7%) of 2829 patients received RT before PSA progression. Median PSA‐PFS and OS were significantly longer in the RT group than in the no‐RT group and the difference was significant in multivariate analyses (HR = 0.44, 95% CI = 0.33‐0.57 and HR = 0.40, 95% CI = 0.27‐0.60, respectively). The occurrence rate of grade ≥2 SLEs was significantly lower in the RT group (2%) than the no‐RT group (9%) and the difference was significant in multivariate analyses (HR = 0.28, 95% CI = 0.10‐0.76). Using PSM analyses, PSA‐PFS and OS remained significantly different (HR = 0.64, 95% CI = 0.46‐0.89 and HR = 0.47, 95% CI = 0.30‐0.72, respectively), between the RT (n = 182) and the no‐RT (n = 182) groups. The difference in OS was significant in the high metastatic burden cohort (HR = 0.55, 95% CI = 0.37‐0.81).

**Conclusions:**

Addition of local RT to standard treatment for de novo metastatic prostate cancer patients tends to have the potential to extend survival, even in patients with high metastatic burden, and to reduce SLEs.

## 1 INTRODUCTION

Prostate cancer is the second leading cause of cancer‐related death in men in western countries^1^ and its incidence in Japan is increasing rapidly.^2^ Between 10% and 15% of prostate cancer patients present with metastatic disease at first diagnosis and typically receive systemic treatment with androgen deprivation therapy (ADT) combined with abiraterone or docetaxel and other androgen receptor axis‐targeted therapies, especially those with a high metastatic burden. Retrospective analyses have noted an association between RT of the primary tumor in patients with metastatic prostate cancer and improved overall survival (OS).^3^, ^4^, ^5^ Therefore, local treatment of the primary tumor in patients with metastatic prostate cancer might be more useful than previously appreciated. The HORRAD trial randomized patients with metastatic prostate cancer to ADT, with or without local RT, and found no evidence of an OS benefit. However, the trial raised the possibility that survival may be improved in a subgroup of patients with fewer than five bone metastases.^6^ The STAMPEDE trial showed no OS benefit of local RT in all metastatic prostate cancer patients; however, a subgroup analysis showed that RT improved the survival in men with a low metastatic burden.^7^

It was reported that the prognosis of prostate cancer patients receiving primary ADT in Japan is significantly better than in western counties.^8^ Therefore, the efficacy of local RT for Japanese patients with metastatic prostate cancer might differ from the previously reported results. Some advanced prostate cancer patients experience local symptoms such as hematuria, urinary retention, or ureteral obstruction during long‐term treatment.^9^ Local treatment might have the potential to prevent these symptomatic local events (SLEs).^10^ However, SLEs such as hematuria or urinary frequency occur secondary to the local RT.^11^ The objective of our retrospective study was to evaluate the efficacy of local RT for extending the survival time and reducing SLEs in de novo metastatic prostate cancer patients with high and low metastatic burdens at institutes participating in the Japan Urologic Oncology Group (JUOG).

## 2 PATIENTS AND METHODS

### 2.1 Study population

This was a retrospective study of patients who were initially diagnosed with metastatic prostate cancer between 2008 and 2017 at 30 university, public, and private hospitals participating in the JUOG. This study was approved by the institutional review board of each institute. The approval number was O‐0403 for Miyazaki University Hospital. All patients had pathologically proven adenocarcinoma of the prostate, and extra‐regional lymph node or distant metastasis was detected by computed tomography (CT) or bone scan at the time of diagnosis. The patient backgrounds and survival data were retrospectively obtained from medical records. In the patients who received local RT during treatment, the date and radiation dose were checked. The treatment start was defined as the date of initial ADT. The state of prostate‐specific antigen (PSA) progression under primary ADT was defined as PSA levels increased by 25% from nadir and higher than 2.0 ng/mL under testosterone levels of <50 ng/dL (1.73 nmol L^−1^). Patients who received radical prostatectomy were excluded. Patients who received RT before PSA progression at a radiation dose of 50 Gy or higher were included in the RT group, and others were included in the no‐RT group. The patients were separated into those with a low metastatic burden (extra regional lymph node metastasis or <4 bone metastases without visceral metastasis) and those with a high metastatic burden (≥4 bone metastases or visceral metastasis). SLEs based on the Common Terminology Criteria for Adverse Events (CTCAE) version 5.0 were defined as cystitis, hematuria, urinary frequency, urinary retention, ureteral obstruction, and urinary tract pain. The type, grade, and occurrence date of the SLEs were evaluated based on medical records.

### 2.2 Statistical analysis

The data are presented as medians and interquartile ranges (IQR). PSA‐progression free survival (PFS) and OS were estimated using the Kaplan‐Meier method. Associations of clinicopathological variables including age ≥73 y, PSA ≥160 ng/mL, hemoglobin (Hb) ≤13 g/dL, lactate dehydrogenase (LDH) ≥200 IU/L, alkaline phosphatase (ALP) ≥300 IU/L, Gleason score (GS) ≥9, prostate volume (PV) ≥50 mL, clinical stage ≥T3b, high metastatic burden, and receiving RT, with PSA‐PFS and OS, were assessed using univariate and multivariate Cox proportional hazards models. The association with the occurrence of grade ≥2 SLEs was assessed by univariate and multivariate logistic regression analyses. Baseline characteristics were compared across groups using Fisher’s exact and Mann‐Whitney tests. To match the patients’ background between the groups, propensity score matching (PSM) was performed using a caliper for neighborhood‐based estimation using the propensity scores calculated by the logistic regression models using the parameters of age, levels of PSA, Hb, LDH and ALP, GS ≥ 9, and high metastatic burden. They were significantly different between the groups and correlated with PSA‐PFS or OS. After PSM, the baseline characteristics data were presented as mean and standard deviation (SD) and compared using the paired *t* test. Thereafter, PSA‐PFS and OS were compared in the low and high metastatic burden cohorts, and interactions for variables of metastatic burden and the effect of local RT on survival were analyzed using Cox proportional hazards models adjusted for stratification factors with emphasis on metastatic burden.^12^ All statistical analyses were performed with EZR (Saitama Medical Center, Jichi Medical University, Saitama, Japan), which is a graphical user interface for R (R Foundation for Statistical Computing, Vienna, Austria). *P *< .05 was considered statistically significant.

## 3 RESULTS

The median follow‐up duration from the start of initial ADT was 36 months (IQR, 19‐61). Among 2823 patients included in this study, 384 (14%) received local RT during the follow‐up duration, of whom 205 (7%) received RT at a dose of 50 Gy or more before PSA progression and were included in the RT group. The median radiation dose in the RT group was 70 Gy (IQR, 70‐74). The primary treatment was ADT alone in 380 (13%), ADT+bicalutamide in 2365 (84%), ADT+docetaxel in 62 (2%), and ADT+abiraterone in one (0.04%).

**FIGURE 1:**
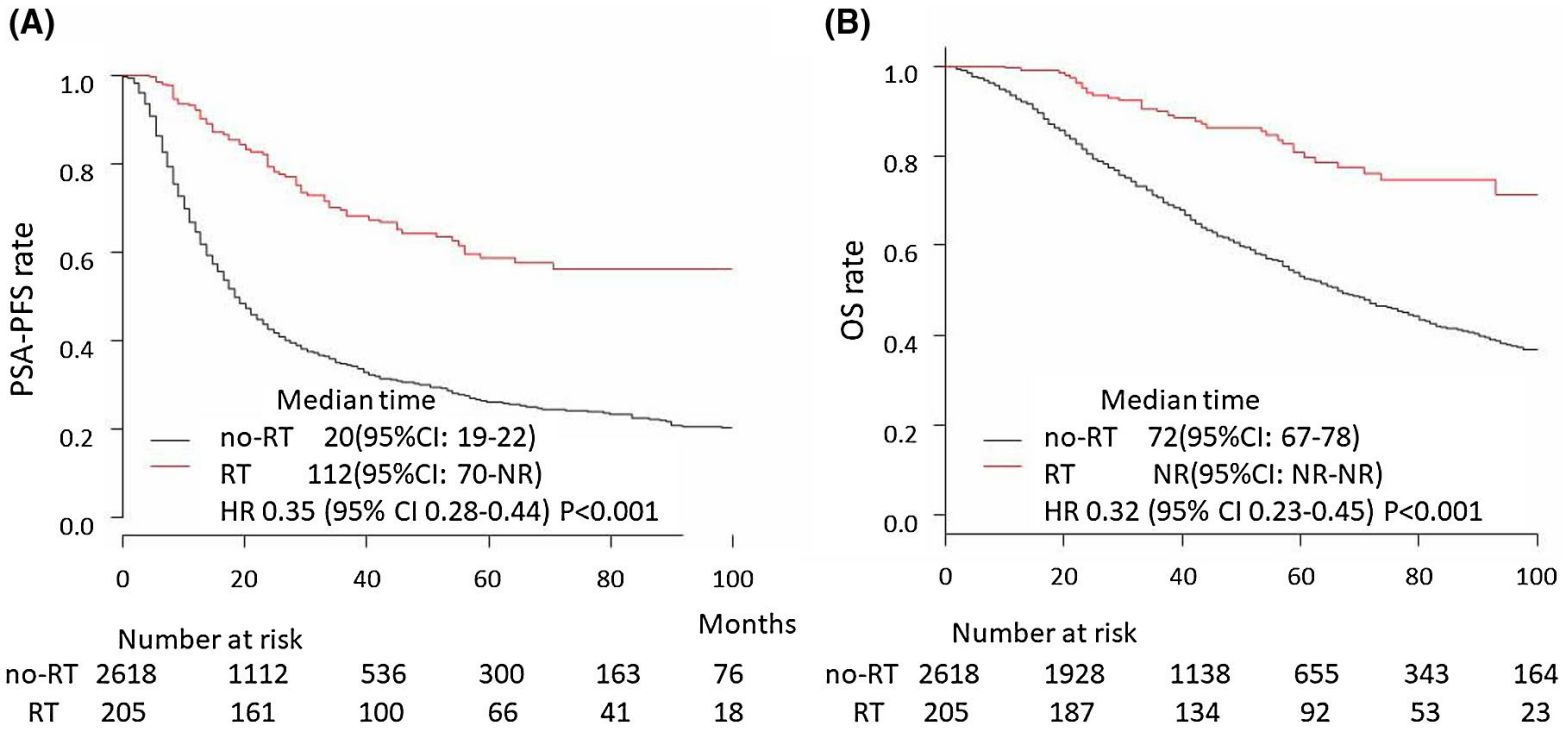
PSA‐PFS and OS after initial hormonal therapy. (A) PSA‐PFS in the all‐patient cohort in the RT group [median = 112 months, 95% CI = 70‐not reached (NR)] and no‐RT group (median = 20 months, 95% CI = 19‐22); HR = 0.35, 95% CI = 0.28‐0.44, *P *< .001. (B) OS in the all‐patient cohort in the RT group (median = NR months, 95% CI = NR‐NR) and no‐RT group (median = 72 months, 95% CI = 67‐78 months); HR = 0.32, 95% CI = 0.23‐0.45, *P *< .001

In total, 1893 patients (57%) had PSA progression under primary treatment and the median PSA‐PFS was 23 months. Between the no‐RT and RT groups, age, levels of PSA, LDH, ALP, Hb, the number of patients with a total GS of 9 or higher, and the number with a high metastatic burden were significantly different (Table 1). PSA‐PFS was significantly longer in the RT group than in the no‐RT group (HR = 0.35, 95% CI = 0.28‐0.44, *P *< .001) (Figure 1A). In univariate Cox proportional hazard analyses, PSA <160 ng/mL, Hb >13 g/dL, LDH <200 IU/L, ALP <300 IU/L, GS <9, clinical stage <T3b, low metastatic burden, and RT+ were significantly associated with longer PSA‐PFS. In multivariate analyses of these parameters, RT+ was still significantly associated with longer PSA‐PFS (HR = 0.44, 95% CI = 0.33‐0.57, *P *< .001) (Table 2).

**TABLE 1:** Patient characteristics

Factors	Total	no‐RT group	RT group*	*P* **
	(n = 2823)	(n = 2618)	(n = 205)	
Median (IQR) age at diagnosis (y)	73 (67‐79)	73 (67‐79)	69 (64‐74)	<.001
Median (IQR) PSA at diagnosis (ng/mL)	226 (58‐786)	241 (63‐839)	85 (22‐305)	<.001
Median (IQR) Hb at diagnosis (g/dL)	13.5 (12.0‐14.5)	13.4 (11.9‐14.5)	14.2 (13.2‐15.0)	<.001
Median (IQR) LDH at diagnosis (IU/L)	203 (175‐249)	205 (176‐252)	185 (161‐215)	<.001
Median (IQR) ALP at diagnosis (IU/L)	332 (235‐682)	343 (239‐717)	278 (210‐369)	<.001
Gleason score (GS) ≥ 9	1683 (63%)	1569 (63%)	114 (56%)	.042
Prostate volume (PV) > 50 mL	701 (29%)	647 (29%)	54 (31%)	.544
cT stage ≥3b	1612 (57%)	1110 (43%)	116 (57%)	.826
High metastatic burden	1790 (63%)	1700 (65%)	90 (44%)	<.001

Abbreviation: IQR, interquartile ranges.

* RT with 50Gy or highger dose befocer CRPC;

** *P*: Mann‐Whitney test or chi square test.

**TABLE 2:** Cox proportional hazard analyzes for PSA progression free survival

Factors	Univaritate		Multivariate	
	Hazard ratio (95%CI)	*P*	Hazard ratio (95%CI)	*P*
Age ≥ 73 y	0.91 (0.83‐1.00)	.048*	0.84 (0.75‐0.93)	.002*
PSA ≥ 160 ng/mL	1.57 (1.43‐1.74)	<.001*	1.21 (1.07‐1.37)	.002*
Hb ≤ 13 g/dL	1.59 (1.44‐1.75)	<.001*	1.31 (1.16‐1.47)	<.001*
LDH ≥ 200 IU/L	1.49 (1.35‐1.65)	<.001*	1.24 (1.11‐1.39)	<.001*
ALP ≥ 300 IU/L	1.84 (1.65‐2.04)	<.001*	1.37 (1.21‐1.55)	<.001*
GS ≥ 9	1.78 (1.60‐1.98)	<.001*	1.67 (1.48‐1.88)	<.001*
PV ≥ 50 mL	1.09 (0.97‐1.22)	.146	1.01 (0.90‐1.14)	.855
≥cT3b	1.32 (1.20‐1.46)	<.001*	1.18 (1.05‐1.32)	.006*
High metastatic burden	1.97 (1.77‐2.19)	<.001*	1.41 (1.24‐1.61)	<.001*
RT+	0.35 (0.28‐0.44)	<.001*	0.44 (0.33‐0.57)	<.001*

* *P* < .05.

The median OS of all patients was 78 months. OS was significantly longer in the RT group than in the no‐RT group (HR = 0.32, 95% CI = 0.23‐0.45, *P *< .001) (Figure 1B). In univariate Cox proportional hazard analyses, age <73 years, PSA <160 ng/mL, Hb >13 g/dL, LDH <200 IU/L, ALP <300 IU/L, GS <9, clinical stage <T3b, low metastatic burden, and RT+ were significantly associated with longer OS. In multivariate analyses including these parameters, RT+ was still significantly associated with longer OS (HR = 0.40, 95% CI = 0.27‐0.60, *P *< .001) (Table 3).

**TABLE 3:** Cox proportional hazard analyzes for overall survival

Factors	Univaritate		Multivariate	
	Hazard ratio (95%CI)	*P*	Hazard ratio (95%CI)	*P*
Age ≥ 73 y	1.25 (1.11‐1.42)	<.001*	1.22 (1.05‐1.41)	.008*
PSA ≥ 160 ng/mL	1.24 (1.09‐1.40)	<.001*	0.82 (0.70‐0.96)	.016*
Hb ≤ 13 g/dL	1.99 (1.75‐2.26)	<.001*	1.51 (1.29‐1.75)	<.001*
LDH ≥ 200 IU/L	2.04 (1.78‐2.33)	<.001*	1.60 (1.38‐1.86)	<.001*
ALP ≥ 300 IU/L	1.89 (1.65‐2.17)	<.001*	1.46 (1.23‐1.72)	<.001*
GS ≥ 9	1.76 (1.53‐2.02)	<.001*	1.78 (1.51‐2.09)	<.001*
PV ≥ 50 mL	0.97 (0.83‐1.12)	.639	0.92 (0.79‐1.09)	.334
≥cT3b	1.18 (1.04‐1.34)	.010*	1.04 (0.90‐1.21)	.569
High metastatic burden	1.87 (1.63‐2.14)	<.001	1.45 (1.21‐1.74)	<.001*
RT+	0.32 (0.23‐0.45)	<.001	0.40 (0.27‐0.60)	<.001

* *P* < .05.

**FIGURE 2:**
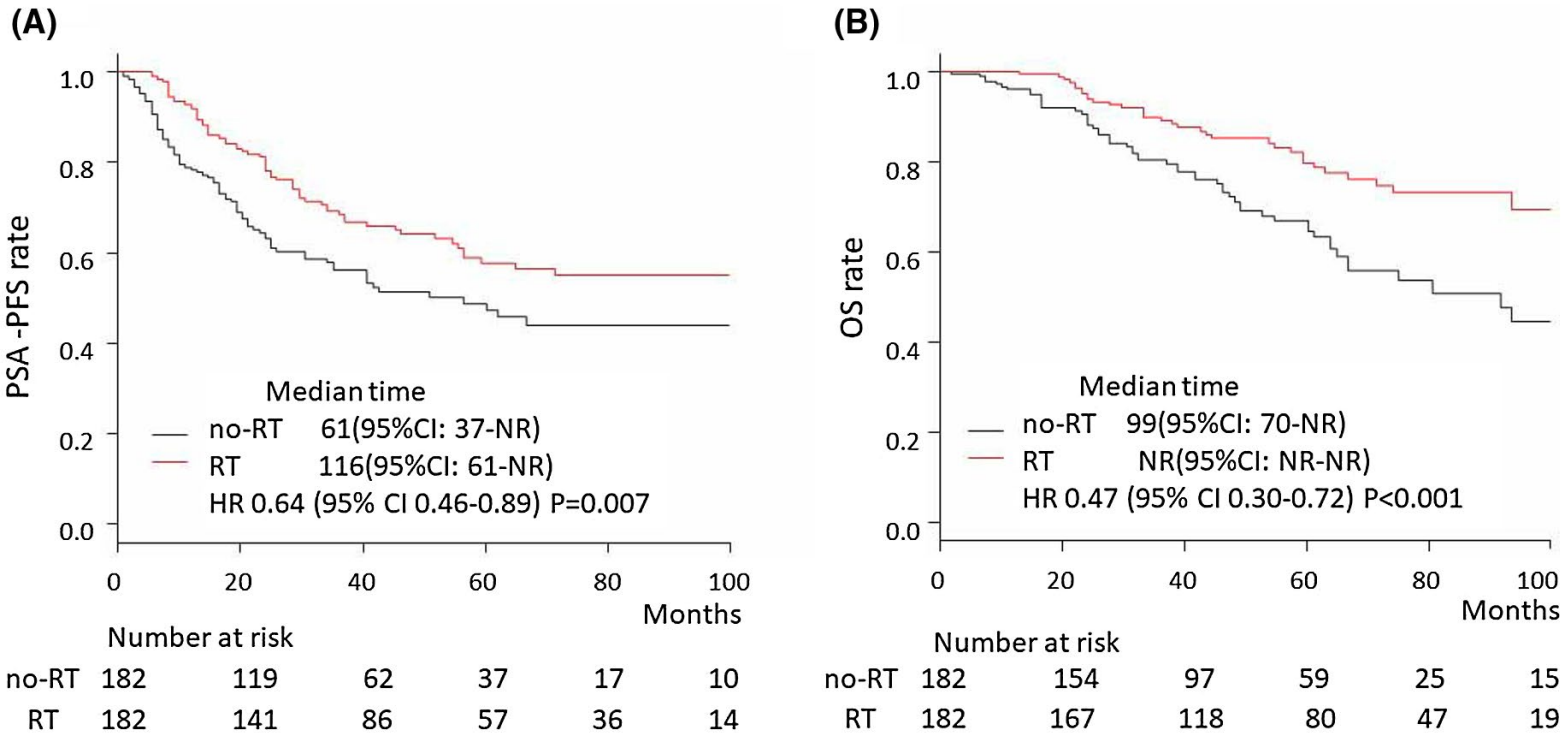
PSA‐PFS and OS after propensity score matching in the all‐patient cohort. (A) PSA‐PFS in the all‐patient cohort in the RT group (median = 116, 95% = CI 61‐NR) and no‐RT group (median = 61 months, 95% CI = 37‐NR); HR = 0.64, 95% CI = 0.46‐0.89, *P* = .007. (B) OS in the all‐patient cohort in the RT group (median = NR, 95% CI = NR‐NR) and no‐RT group (median = 99 months, 95% CI = 70‐NR); HR = 0.47, 95% CI = 0.30‐0.72, *P *< .001

**FIGURE 3:**
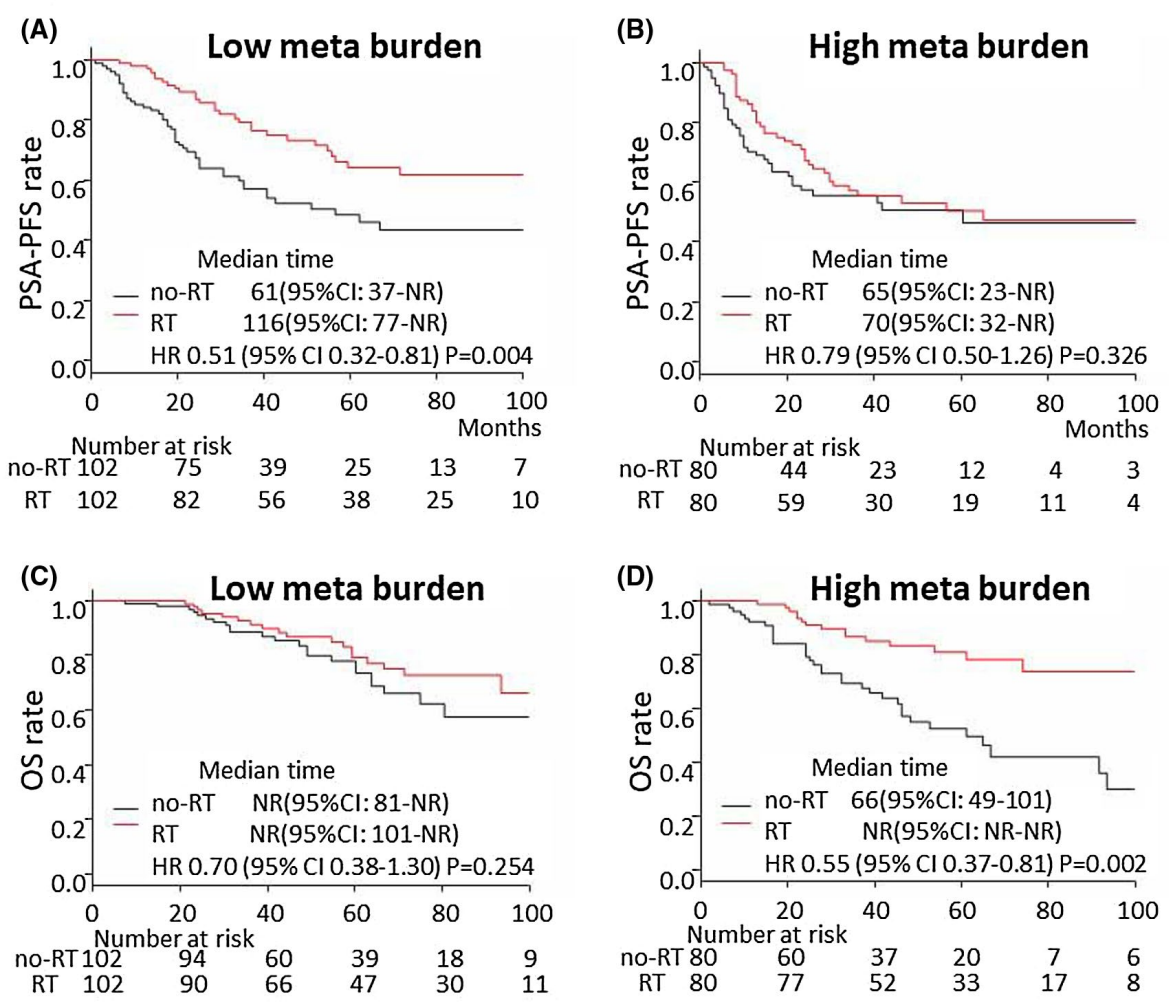
OS after propensity score matching in the all‐patient cohort, high metastatic burden cohort, and low metastatic burden cohort. (A) PSA‐PFS in the low metastatic burden cohort in the RT group (median = 116, 95% CI = 77‐NR) and no‐RT group (median = 61 months, 95% CI = 37‐NR); HR = 0.51, 95% CI = 0.32‐0.81 months, *P* = .004. (B) PSA‐PFS in the high metastatic burden cohort in the RT group (median = 70, 95% CI = 32‐NR) and no‐RT group (median = 65 months, 95% CI = 23‐NR); HR = 0.79, 95% CI = 0.50‐1.26, *P* = .326. (C) OS in the low metastatic burden cohort in the RT group (median = NR, 95% CI = 101–NR) and no‐RT group (median = NR months, 95% CI = 81‐NR), HR = 0.70, 95% CI = 0.38‐1.30 months, *P* = .254. (D) OS in the high metastatic burden cohort in the RT group (median NR, 95% CI NR‐NR) and no‐RT group (median = 66 months, 95% CI = 49‐101); HR = 0.55, 95% CI = 0.37‐0.81, *P* = .002

To reduce the selection bias between the no‐RT and RT groups, PSM was performed. The background of the patients was not significantly different between the no‐RT group (n = 182) and the RT group (n = 182) after PSM (Table 4). PSA‐PFS and OS were significantly longer in the RT group than in the no‐RT group (HR = 0.64, 95% CI = 0.46‐0.89, *P* = .007 and HR = 0.47, 95% CI = 0.30‐0.72, *P *< .001, respectively) (Figure 2). After PSM, PSA‐PFS and OS were compared in the high and low metastatic burden groups. The difference in PSA‐PFS was more significant in the low metastatic burden cohort (HR = 0.51, 95% CI = 0.32‐0.81, *P* = .004) than in the high metastatic burden cohort (HR = 0.79, 95% CI = 0.50‐1.26) (Figure 3A,B). The difference in OS was more significant in the high metastatic burden cohort (HR = 0.55, 95% CI = 0.37‐0.81) than in the low metastatic burden cohort (HR = 0.70, 95% CI = 0.38‐1.30) (Figure 3C,D). In the interaction analyses, there was no significant heterogeneity in the local RT effect on PSA‐PFS between the low and high metastatic burden cohorts (interaction, *P* = .229). A significant benefit of local RT on OS was found in the high metastatic burden cohort compared with the low metastatic burden cohort (interaction, *P* = .049).

**TABLE 4:** Background of patients in RT and no‐RT groups after prpoensity score matching

Factors	no‐RT (182)	RT (182)	*P* *
Mean (SD) age (y)	68 (8)	68 (8)	.741
Mean (SD) PSA (ng/mL)	444 (900)	444 (1121)	.999
Mean (SD) Hb (g/dL)	14.2 (1.6)	14.0 (1.6)	.228
Mean (SD) LDH (IU/L)	195 (62)	194 (57)	.900
Mean (SD) ALP (IU/L)	510 (915)	528 (1309)	.878
GS ≥ 9	108 (59%)	103 (57%)	.886
High metastatic burden	80 (44%)	80 (44%)	.940

Note

SD: standard deviation.

* *P*: paired *t* test or chi square test.

The occurrence rate of each SLE was cystitis in 42 patients (1%), hematuria in 135 (5%), urinary frequency in 99 (4%), urinary retention in 147 (5%), ureteral obstruction in 61 (2%), urinary tract pain in 35 (1%), and others in 154 (5%). In total, the occurrence rate of any‐grade SLEs was 11% and that of grade ≥2 SLEs was 8% during follow‐up. The median time from ADT initiation to the occurrence of any‐grade and grade ≥2 SLEs was 20 (IQR, 10‐41) and 22 (IQR, 12‐44) months, respectively. The occurrence rate of grade ≥2 SLEs was significantly lower in the RT group (4/205, 2%) than in the no‐RT group (224/2618, 9%) (*P *< .001), although the median follow‐up was significantly longer in the RT group than in the no‐RT group (56 and 34 months, respectively; *P *< .001). In univariate logistic regression analyses, GS ≥9, PV ≥50 mL, and clinical stage ≥T3b were positively associated with the occurrence of grade ≥2 SLEs, while ALP ≥300 IU/L and RT+ were negatively associated. In multivariate analyses including these parameters, RT+ was still significantly associated with a lower rate of grade ≥2 SLEs (HR = 0.28, 95% CI = 0.10‐0.76, *P* = .013) (Table 5). After PSM, the occurrence rate of grade ≥2 SLEs was still significantly lower in the RT group (4/182, 2%) than in the no‐RT group (26/182, 14%) (*P *< .001).

**TABLE 5:** Logistic regression analyses for grade ≥2 symptomatic local event occurrence

Factors	Univaritate		Multivariate	
	Hazard ratio (95%CI)	*P*	Hazard ratio (95%CI)	*P*
Age ≥ 73 y	0.86 (0.66‐1.13)	.276	0.93 (0.67‐1.30)	.131
PSA ≥ 160 ng/mL	0.77 (0.659‐1.01)	.062	0.79 (0.55‐1.13)	.193
Hb ≤ 13 g/dL	1.04 (0.78‐1.40)	.775	1.03 (0.72‐1.47)	.876
LDH ≥ 200 IU/L	1.05 (0.79‐1.41)	.720	0.98 (0.71‐1.38)	.927
ALP ≥ 300 IU/L	0.70 (0.53‐0.94)	.017*	0.66 (0.46‐0.95)	.026*
GS ≥ 9	1.37 (1.01‐1.85)	.040*	1.49 (1.04‐2.14)	.030*
PV ≥ 50 mL	1.62 (1.19‐2.20)	.002*	1.50 (1.06‐2.11)	.020*
≥cT3b	1.75 (1.31‐2.35)	<.001*	1.57 (1.10‐2.22)	.012*
High metastatic burden	1.02 (0.77‐1.35)	.916	1.07 (0.74‐1.56)	.724
RT+	0.21 (0.08‐0.58)	.002*	0.28 (0.10‐0.76)	.013*

* *P* < .05.

## 4 DISCUSSION

Recent retrospective studies have used national databases to evaluate the outcome of local treatment such as radical prostatectomy or local RT for metastatic prostate cancer.^3^, ^4^, ^5^ They have reported that local treatment yields lower cancer‐specific mortality rates when combined with ADT than when local treatment is not given. Data from several studies suggest that patients with metastatic disease derive the most benefit from local treatment when they have more favorable tumor characteristics. Löppenberg et al. performed a multivariable Cox regression analysis of 15 501 metastatic prostate cancer patients in the National Cancer Database and found that patients with a low tumor burden and a good general health status appeared to benefit most from local treatment.^13^ Pompe et al. used data from 13 906 metastatic prostate cancer patients in the Surveillance, Epidemiology, and End Results (SEER) database. They found that local treatment had significant benefits in patients with M1a and M1b with low PSA levels but no benefit in patients with M1b with high PSA levels and M1c.^14^ In our multi‐institutional retrospective study using the medical records of 2823 de novo metastatic prostate cancer patients, local RT was significantly associated with longer PSA‐PFS (HR = 0.44, 95% CI = 0.33‐0.57) and OS (HR = 0.40, 95% CI = 0.27‐0.60) in multivariate Cox proportional hazard analyses. Moreover, PSA‐PFS and OS were significantly different in PSM analyses. The effect of local RT on PSA‐PFS was more significant in the low metastatic burden cohort than in the high metastatic burden cohort. These results are consistent with previous reports. However, a survival benefit of local RT was found in the high metastatic burden cohort (HR = 0.55, 95% CI = 0.37‐0.81) and not in the low metastatic burden cohort (HR = 0.70, 95% CI = 0.38‐1.30). These results are not consistent with the results of PSA‐PFS or previous reports.

The HORRAD study was the first randomized controlled trial (RCT) in patients with metastatic prostate cancer (n = 432) to evaluate local RT combined with ADT compared with ADT alone, with OS as the primary endpoint.^6^ No OS benefit was shown for local RT after a median follow‐up of 47 months. However, OS tended to increase after the addition of RT in a subgroup of patients with fewer than five bone metastases in the forest plot data. The STAMPEDE study was the next RCT of patients with metastatic prostate cancer (n = 2061), which showed that local RT did not improve OS for unselected patients (HR = 0.92, 95% CI = 0.80‐1.16).^7^ However, a prespecified analysis showed that local RT improved OS in patients with low metastatic burden (HR = 0.68, 95% CI = 0.52‐0.90). Local RT could be a standard treatment option for metastatic prostate cancer patients with a low metastatic burden but not for those with a high metastatic burden. These results were not consistent with our study. This variation might be partly caused by the difference in the definition of high metastatic burden. In these two studies, the median OS of the control group was 45 and 46 months, respectively. In our study, the median OS of the control group was 72 months, which was significantly longer than the two published studies. The difference in the patient background might affect the difference in OS among the studies. Cooperberg et al reported that men treated in Japan tend to be diagnosed at an older age and with more advanced tumors than men treated in the USA. However, both cancer‐specific survival and OS were substantially better for men treated in Japan compared with the USA, even after adjusting for disease risk, patient characteristics, and type of ADT.^8^ The reasons behind these substantial differences probably include both genetic and dietary/environmental factors.^15^ Moreover, patients with extremely poor prognoses were excluded after PSM. The longer OS of patients with metastatic prostate cancer in this study might have contributed to our results showing that local RT had a survival benefit even in patients with a high metastatic burden. And the OS of patients with a low metastatic burden was so long that local RT had benefitted only PSA‐PFS and not OS. The difference in the prognosis of patients between Japan and other countries should be considered when determining the indications for local RT in metastatic prostate cancer patients. These results indicate that the addition of local RT to the standard treatment for metastatic prostate cancer has the potential to extend survival not only in patients with a low metastatic burden but also in some patients with a high metastatic burden.

It was reported that SLEs in prostate cancer patients are directly associated with quality of life (QOL), and appropriate interventions for end‐of‐life adverse events, including SLE, improve QOL.^16^, ^17^ Arai et al. evaluated the pattern of prostate cancer progression in patients receiving ADT and reported that local progression was observed in 46% of patients with stage N1 or M1 disease.^18^ We previously reported that major SLEs that required therapeutic intervention were observed in 28% of patients who died of prostate cancer.^9^ Local RT was effective for the palliation of these SLEs.^19^ Therefore, prophylactic local RT might have the potential to reduce the occurrence of these SLEs to prevent local progression. However, local RT might increase some treatment‐related SLEs.^11^ The STAMPEDE study demonstrated that the number of patients with one or more symptomatic local event did not differ between the RT and the control groups.^7^ However, in our study, the occurrence rate of grade ≥2 SLEs was significantly lower in the RT group (4/205, 2%) than in the no‐RT group (224/2618, 9%) (*P *< .001). In a previous retrospective study, local treatment by radical prostatectomy or RT significantly reduced the incidence of SLEs compared with no primary treatment (33% vs 55%, *P* = .001).^10^ The occurrence rate of SLEs in our study was lower than that in the previous studies, probably because the data were retrospectively collected from medical records and the median follow‐up was only 37 months, including recently diagnosed patients. Patients who have lived for a long time and have received long‐term ADT might be at the highest risk of SLEs.^9^ In our study GS ≥9, PV ≥50 mL, and ≥cT3b were significantly associated with high SLE rate in multivariate analyses. Metastatic prostate cancer patients harboring these risk factors might be good candidates for local RT, and the precise evaluation of clinical T stage by magnetic resonance imaging (MRI) may be useful in identifying good candidates. Local RT might have the potential to prevent the occurrence of SLEs in patients with local progression and maintain better QOL, especially for metastatic prostate cancer patients with good prognosis. In our study, the radiation dose differed among institutes and the median dose was 70 Gy. In another RCT of local RT with neoadjuvant ADT for localized prostate cancer, higher‐dose RT (74 Gy) had better progression‐free survival than standard dose RT (64 Gy).^20^ The applied radiation dose in the HORRAD study was 70 Gy/35 fractions and that in the STAMPEDE study was 55 Gy/20 fractions or 36 Gy/six fractions, which was less than 67 Gy delivered in standard fractionation. To evaluate the efficacy of local RT for reducing local progression and concomitant SLEs, an RCT with and without RT at higher doses is needed, especially for locally advanced prostate cancer.

There are some limitations in the present study. First, this was a retrospective multi‐institutional study and the selection bias for local treatment might have been strongly associated with the better OS in the RT group. The indication and timing of RT differed among the institutes. There were no data about whether metastasis‐directed radiation therapy was performed . Even in the multivariate Cox proportional hazard and PSM analyses, all parameters correlating with prognosis, such as performance status or comorbidity of the patients, were not included. Second, the definition of high and low metastatic burden was based on CT and bone scintigraphy according to the CHAARTED study.^21^ The precise region of bone metastasis could not be revealed in many patients. Therefore, we used the definition of high metastatic burden as four or more bone metastases or visceral metastasis, instead of the definition used in the CHAARTED and STAMPEDE studies as four or more lesions with at least one outside the axial skeleton. Novel imaging modalities such as diffusion‐weighted whole‐body MRI or prostate‐specific membrane antigen/positron emission tomography may be helpful in evaluating metastatic volume in the future to determine the indications for local RT treatment. Third, most patients in this study received ADT alone or ADT+bicalutamide as a primary treatment. It might cause the discrepancy between the effect of local RT on PSA‐PSA and OS in high metastatic burden cohort. Recently, ADT combined with docetaxel, abiraterone, or other androgen receptor axis‐targeted therapies have been considered standard primary treatment options for metastatic prostate cancer patients. The value of local RT for patients receiving abiraterone is being tested in the PEACE1 trial.^22^ Further RCTs are needed to evaluate the efficacy of local RT under the various therapies for patients, especially those with a high metastatic burden.

In conclusion, our retrospective multi‐institutional study indicated that addition of local RT to the standard treatment for metastatic prostate cancer has the potential to reduce SLEs and prolong patient survival. Local RT might be a treatment option for selected patients with metastatic prostate cancer.

## CONFLICT OF INTEREST

None
